# A Cross-Sectional Study on the Psychological Impact of Stress, Anxiety and Depressive Symptoms in COVID-19 Patients

**DOI:** 10.7759/cureus.27724

**Published:** 2022-08-05

**Authors:** Kiran Jakhar, Khalid Y Lone, Rakesh Gupta, Anurag Srivastava, Hariom K Solanki

**Affiliations:** 1 Department of Psychiatry, Government Institute of Medical Sciences​, Greater Noida, IND; 2 Internal Medicine, Additional Primary Health Centre, Bazpur, IND; 3 Department of Pediatrics, Government Institute of Medical Sciences​, Greater Noida, IND; 4 Department of Community Medicine, Government Institute of Medical Sciences​, Greater Noida, IND

**Keywords:** depressive symptoms, anxiety, ptsd, psychological impact, covid-19, sars-cov2

## Abstract

Background

COVID-19 has become a public health emergency caused by a negative-stranded ribonucleic acid (RNA) virus, which previously caused severe acute respiratory syndrome (SARS) and the Middle East respiratory syndrome (MERS). In addition, the pandemic led to an array of social, financial, psychological, and psychiatric issues.

Methods

An observational study was performed with consecutive sampling and included patients diagnosed with COVID-19 and admitted to the hospital. Subjects were evaluated using a semi-structured proforma and an online survey of the revised Impact of Event Scale (IES-R) 15 days post-discharge from the hospital.

Results

A total of 201 subjects were recruited, of which approximately 30% were female, and the rest were male. Approximately half of the subjects experienced symptoms suggestive of post-traumatic stress disorder (PTSD), with the highest number of patients falling in the severe category. Also, females experienced more anxiety symptoms than males (p=0.002).

Conclusion

Infection with COVID-19 and hospitalization tended to significantly impact individuals' mental state. In addition, the majority of subjects had severe symptoms of post-COVID PTSD, warranting the need for treatment.

## Introduction

COVID-19 is a one-of-a-kind pandemic and public health emergency due to its high infectivity and mortality rate. Coronavirus is a negative-stranded RNA virus that causes infections ranging from a common cold to severe acute respiratory syndrome (SARS) [1]. The virus has previously been implicated in neuropsychiatric illness during and after SARS and Middle East Respiratory Syndrome (MERS) outbreaks [2]. The diagnosis of various psychiatric illnesses has been shown to increase within a follow-up period of 1-50 months in SARS-infected persons, where 27.1% had chronic fatigue syndrome [3-5]. The current prevalence of any psychiatric disorder at 30 months post-SARS was 33.3%. Post-traumatic stress disorder (PTSD) (25.6%) was the most prevalent long-term psychiatric condition, which was found in one-fourth of the patients (25.6%), followed by depressive disorders (15.6%) [6]. 

The data suggest that COVID-19 might induce psychopathological sequelae through direct viral infiltration of the CNS or indirectly via immune response [3], leading to delirium, depression, anxiety, and insomnia [2]. There is a pathophysiological mechanism underlying mood disorders, where psychosis and anxiety disorders result from the interaction between infection-triggered disturbance of the innate and adaptive immune system and neurotransmitters [7].

COVID-19 is associated with various psychosocial changes leading to travel restrictions in the form of government-imposed lockdown periods. These include social isolation, cancellation of religious, sports, cultural, and entertainment events, fear of contracting disease, quarantine rules, loss of support from the family, economic setback, asymptomatic incubation period, shortage of medical protective supplies, medical staff, and hospital beds, and overabundance of (mis)information on social media leading to anger, loneliness, boredom, anxiety, fatigue, and emotional distress [8].

With SARS and PTSD, anxiety and depression were reported during the epidemic [9], and was also noticed after one month [10], one year [11], and 30 months and beyond [6]. The virus responsible for COVID-19 belongs to the same viral family as SARS and MERS.

A study was conducted in China regarding psychological impact, anxiety, depression, and stress during the initial stage of the COVID-19 outbreak using the revised Impact of Event Scale (IES-R) and the Depression, Anxiety, and Stress Scale (DASS-21) on the general population. It showed that more than half of the sample of 1210 individuals (53.8%) rated their psychological impact as moderate to severe [12].

A study by Li S et al. regarding the impact of COVID-19 on mental health showed that negative emotions like anxiety, depression, indignation, and sensitivity to social risks increased. In contrast, scores of positive emotions and life satisfaction decreased. Thus, it is necessary to evaluate the psychological impact of COVID-19 on patients who are or were suffering from the infection where the burden may be immense [13].

A study by Vlake JH et al. in the Netherlands in 2021 reported that 16% and 13% of 294 patients reported probable PTSD, 29% and 20% probable anxiety, and 32% and 24% probable depression at one and three months after hospital discharge, respectively, as assessed on IES-R and Hamilton Anxiety and Depression Scale (HADS) [14].

Thus, there is immense data to suggest that various psychiatric disorders are associated with COVID-19 infection. So, we hypothesized that particularly admission with COVID-19 increases the psychological impact of COVID-19. Therefore, the current study specifically aims to assess the burden of psychological impact (i.e., stress, anxiety, and depressive symptoms) in COVID-19 admitted patients and determine the relationship between the impact of sociodemographic and clinical parameters if any.

## Materials and methods

An observational study with consecutive sampling was done at one of the tertiary care centers of Western Uttar Pradesh. The data collection was done for six months, from May 2020 to October 2020. All the patients of COVID-19 who were admitted at index COVID designated tertiary health care center in Western Uttar Pradesh were included. Approval from the GIMS Institutional Ethical Committee (reference no. GIMS/IEC/HR/EFR/2020/08) was given at the outset. All patients diagnosed with COVID-19, willing to participate, and who agreed to give written informed consent were included in the study. At the start of the study, the participants were informed briefly about its purpose.

Inclusion criteria were both genders, aged above 18 years, and; all patients diagnosed with COVID-19 and willing to give written informed consent. Exclusion criteria were lifetime substance use disorder (excluding tobacco and caffeine) or any psychiatric disorder in the past or current as per the Diagnostic and Statistical Manual-5 (DSM-5), and pregnant and lactating women.

The sample size included all COVID-19 patients admitted to the hospital for a duration of six months, from May 2020 to October 2020. The universe of the study was that all the patients of COVID-19 who were admitted at COVID-designated tertiary health care centers in Western Uttar Pradesh were included. After giving informed consent, the subjects were evaluated using a semi-structured proforma designed to collect information regarding sociodemographic variables and an online survey devised for the study. The online survey was given at least 15 days after the discharge from the hospital, which used the IES-R [15], comprising 22 questions to be answered on a Likert scale of 0-4 as follows: 0- Not at all, 1- A little bit, 2- Moderately, 3- Quite a bit, and 4- Extremely.

The IES-R is a short, easily administered self-report questionnaire that is an appropriate instrument to measure the subjective response to a specific traumatic event, using a response set of intrusion, avoidance, hyperarousal, and total subjective stress IES-R score. Scores higher than 24 were considered concerning. The scale's three-factor structure, which includes intrusion, avoidance, and hyperarousal, has adequate internal consistency for each subscale. There is concurrent and discriminative validity, as well as the absence of social desirability effects (Table 1) [16].

**Table 1:** Cut-off value for severity of PTSD symptoms. PTSD: Post-traumatic stress disorder.

Cut-off Range	Severity of PTSD
24-32	PTSD is of clinical concern. Those with these scores do not have full PTSD but will have partial PTSD or at least some of the symptoms.
33-38	Represents the best cut-off for a probable diagnosis of PTSD.
39 and above	High enough to suppress immune system function (even 10 years after an impact event).

The intrusion subscale comprises item numbers: 1, 2, 3, 6, 9, 14, 16, and 20. The avoidance subscale comprises item numbers: 5, 7, 8, 11, 12, 13, 17, and 22. The hyperarousal subscale comprises item numbers: 4, 10, 15, 18, 19, and 21

The data were analyzed using Statistical Package for Social Sciences (SPSS) version 26 after being entered into a Microsoft Excel sheet. A summary of the data is presented as frequencies and percentages for categorical data, while the mean (SD) or mean (interquartile range [IQR]) is used for continuous data depending upon their distribution type. Associations were tested using Chi-squared or Fisher's exact tests for categorical variables. A p-value of <0.05 was considered statistically significant.

## Results

Among patients infected with COVID-19 and admitted to the hospital, a total of 201 subjects were recruited in the study who replied to the online survey.

Out of 201 subjects recruited in the study, the majority were male, accounting for approximately two-thirds of the sample, while one-third were females. Most of the subjects were in the 20-40 years age group, corresponding to the patients infected with COVID-19 in the first wave in India, as the working population got infected first. The education of most of the subjects was at least bachelor's level or its equivalent, and the majority were employed (81.5% of the sample) (Table 2).

**Table 2:** Sociodemographic variables of the study subjects.

S. No.	Parameters	Male n (%)	Female (n%)	Total ( n%)
1.	Gender	142	59	201
2.	Age distribution			
	<20 years	11 (5.47)	4 (1.99)	15 (7.46)
	20-40 years	98 (48.75)	46 (22.88)	144 (71.64)
	40-60 years	25 (12.43)	5 (2.48)	30 (14.92)
	>60 years	8 (3.98)	4 (1.99)	12 (5.97)
3.	Education			
	Up to High School	18 (8.95)	5 (2.48)	23 (11.44)
	Bachelor or equivalent	72 (35.82)	32 (15.92)	104 (51.74)
	Master or equivalent	52 (25.87)	22 (10.94)	74 (36.81)
4.	Employment			
	Employed	125 (62.18)	39 (19.40)	164 (81.5)
	Unemployed	7 (3.48)	3 (1.14)	10 (4.97)
	Student	9 (4.47)	8 (3.98)	17 (8.45)
	Housewife	00 (0)	10 (4.97)	10 (4.97)

Approximately half of the subjects were found to have an impact on their mental health (i.e., having the symptoms of PTSD) (50.73%). In this group, the majority were in a severe category, meaning immune response was suppressed, followed by 18.9% in the mild category. The fewest participants had an average score (Table 3).

**Table 3:** Categorization of subjects according to the cut-off value of the IES-R scale. IES-R: Revised Impact of Event Scale; PTSD: Post-traumatic stress disorder.

IES-R score	Category	Number	Percentage	Interpretation
No illness	<24	99	49.23%	No PTSD
Mild	24-32	38	18.9%	Partial PTSD symptoms
Moderate	33-38	17	8.45%	Probable PTSD symptoms
Severe	≥39	47	23.38%	Immune response suppressed

Table 4 provides a summary of individual item responses from the study subjects and a summary of scores by the three subscales, which constitute the study tool used in this work. Study items numbers 4, 6, 19, and 20 had a mean score of <1 and a median score of 0 (the same as item number 15), while item number 11 had a relatively higher mean score of 1.67. All other scores were between 1 and 1.4. Overall, it seemed that the subjects in our study were more likely to use avoidance as a coping method than others.

**Table 4:** Mean, median and range of each item of the IES-R scale. IES-R scale: Revised Impact of Event Scale.

S. N+M3+J9:N13	Scale question	Mean (SD)	Median (IQR)	Range
1	Any reminder brought back feelings about it	1.33 (1.32)	1 (0-2)	0-4
2	I had trouble staying asleep	1.04 (1.29)	1 (0-2)	0-4
3	Other things kept making me think about it	1.24 (1.29)	1 (0-2)	0-4
4	I felt irritable and angry	0.94 (1.18)	0 (0-2)	0-4
5	I avoided letting myself get upset when I thought about it or was reminded of it.	1.18 (1.36)	1 (0-2)	0-4
6	I thought about it when I didn’t mean to	0.92 (1.34)	0 (0-2)	0-4
7	I felt as if it hadn’t happened or wasn’t real	1.13 (1.30)	1 (0-2)	0-4
8	I stayed away from reminders of it	1.22 (1.35)	1 (0-2)	0-4
9	Pictures about it popped into my mind	1.21 (1.38)	1 (0-2)	0-4
10	I was jumpy and easily startled	1.22 (1.28)	1 (0-2)	0-4
11	I tried not to think about it	1.67 (1.52)	1 (0-3)	0-4
12	I was aware that I still had a lot of feeling about it, but I didn’t deal with them	1.04 (1.27)	1 (0-2)	0-4
13	My feelings about it were kind of numb	1.17 (1.25)	1 (0-2)	0-4
14	I found myself acting or feeling like I was back in that time	1.03 (1.26)	1 (0-2)	0-4
15	I had trouble falling asleep	1.08 (1.38)	0 (0-2)	0-4
16	I had waves of strong feelings about it	1.32 (1.43)	1 (0-2)	0-4
17	I tried to remove it from my memory	1.38 (1.55)	1 (0-3)	0-4
18	I had trouble concentrating	1.15 ((1.37)	1 (0-2)	0-4
19	Reminders of it caused me to have physical reactions such as sweating, trouble breathing, nausea, or a pounding heart	0.81 (1.22)	0 (0-1)	0-4
20	I had dreams about it	0.70 (1.16)	0 (0-1)	0-4
21	I felt watchful and on guard	1.23 (1.36)	1 (0-2)	0-4
22	I tried not to talk about it	1.29 (1.44)	1 (0-2)	0-4
23	Avoidance subscale (Item nos. 5,7,8,11,12,13,17,22)	10.10 (8.07)	9 (3-16)	0-32
24	Hyperarousal subscale (Item nos. 4,10,15,18,19,21)	6.43 (5.85)	5 (2-10)	0-24
25	Intrusion subscale (Item nos. 1,2,3,6,9,14,16,20)	8.80 (7.71)	7 (3-13)	0-32

In the avoidance subscale, "I tried not to think about it" and "I tried to remove it from my memory" were the highest scored items among patients, while others tried not to talk about it or stayed away from its reminders. These are the most common responses to PTSD, which form its core symptoms. "Any reminder brought back feelings about it" and "I had waves of strong feelings about it" were the highest scored items in the intrusion subscale, while the "I had dreams about it" item had a very low score, signifying few people experienced it. Finally, "I was jumpy and easily startled" and "I felt watchful and on guard" were the highest scored items in the hyperarousal subscale (Table 5). 

**Table 5:** Mean and SD for each item of IES-R Scale. IES-R scale: Revised Impact of Event Scale.

S. No.	Subscale	Mean	SD
Intrusion Subscale			
1.	Any reminder brought back feelings about it	268/201=1.333	1.316
2.	I had trouble staying asleep	209/201=1.039	1.287
3.	Other things kept making me think about it	250/201=1.243	1.286
4.	I thought about it when I didn’t mean to	185/201=0.920	1.159
5.	Pictures about it popped into my mind	244/201=1.213	1.381
6.	I found myself acting or feeling like I was back in that time	207/201=1.029	1.264
7.	I had waves of strong feelings about it	265/201=1.318	1.434
8.	I had dreams about it	140/201=0.696	1.162
Avoidance Subscale			
1.	I avoided letting myself get upset when I thought about it	238/201=1.184	1.360
2.	I felt as if it hadn’t happened or wasn’t real	228/201=1.134	1.302
3.	I stayed away from reminders of it	246/201=1.223	1.354
4.	I tried not to think about it	335/201=1.666	1.517
5.	I was aware that I still had a lot of feeling about it, but I didn’t deal with them	209/201=1.039	1.268
6.	My feelings about it were kind of numb	236/201=1.174	1.250
7.	I tried to remove it from my memory	278/201=1.383	1.554
8.	I tried not to talk about it	260/201=1.293	1.445
Hyperarousal Subscale			
1.	I felt irritable and angry	189/201=0.940	1.181
2.	I was jumpy and easily startled	246/201=1.223	1.278
3.	I had trouble falling asleep	217/201=1.079	1.379
4.	I had trouble concentrating	231/201=1.149	1.373
5.	Reminders of it caused me to have physical reactions such as sweating, trouble breathing, nausea, or a pounding heart	162/201=0.805	1.223
6.	I felt watchful and on guard	247/201=1.228	1.335

As per the data, females tended to experience more anxiety symptoms at different grades than males, which was statistically significant (p=0.002). However, there was no statistically significant difference in categorization with different anxiety grades. However, moderate or severe anxiety was still in higher proportion in females compared to males, with a p-value that was nearly significant (Table 6). The sample size in the study or a more pronounced effect might have turned out to be statistically significant.

**Table 6:** Association of reported anxiety with gender.

	Female	Male	P-value
No anxiety reported	18	81	0.002
Anxiety reported	40	62	
No or mild anxiety	34	103	0.064
Moderate or severe anxiety	24	40	

## Discussion

The uncertain and unpredictable COVID-19 pandemic has globally affected each and every section of society across nearly all dimensions, including education, financial, occupational, and psychological [8]. Every individual was impacted due to COVID-19 regardless of infection status. Those infected with COVID-19 had health issues such as fever, cough, weakness, malaise, breathing difficulty (often necessitating requirement of respiratory support), and even death, while those not infected constantly feared contamination. Therefore, the current study aimed to determine the psychological impact of COVID-19 on patients admitted to the hospital, as this group experienced the fear of succumbing to death due to infection and suffered unprecedented family isolation.

The majority of the 201 subjects recruited in the study were male (two-thirds). The most probable reason for males outnumbering females in the study sample might be that in the first wave of COVID-19, the most affected population was the working class, who had to go out to work as lockdowns were imposed. Most of the subjects were in the age group of 20-40 years, which was the age of the patients infected with COVID-19 in the first wave in India, as the working population got infected first. The subjects were mostly educated (i.e., bachelor's or equivalent), so they may be more agreeable to online surveys and responded in more significant numbers. The majority of the subjects were employed (81.5% of the sample).

Approximately 50.73% of the subjects experienced symptoms of PTSD. Out of which, the highest number of patients fell into the severe category, followed by 18.9% in the mild category. There are studies that bear concurrence with this outcome. For example, a study of the general population of Spain by Parrado-González A and León-Jariego JC in 2020 showed that 24.7% of patients reported moderate-to-severe psychological impact, and 48.8% showed mental health problems during COVID-19, according to the 12-Item General Health Questionnaire (GHQ-12) and IES-R [17].

A study by Islam MS et al. in 2020 investigated the emergence of psychological issues during COVID-19 in university students of Bangladesh using the DASS-21 scale, where depression, anxiety, and stress was 76.1%, 71.5%, and 70.1% for mild symptoms, 62.9%, 63.6%, and 58.6% for moderate symptoms, 35.2%, 40.3%, and 37.7% for severe symptoms, and 19.7%, 27.5%, and 16.5% for very severe symptoms, respectively [18]. In 2020, Alkhamees AA et al. reported that 23.6% of the general population of Saudi Arabia had a moderate-to-severe psychological impact during COVID-19, while 28.3%, 24%, and 22.3% reported moderate-to-severe depressive, anxiety, and stress symptoms under the IES-R and DASS-21 scale [19]. A 2020 study by Choi EP et al. on the general population of Hong Kong reported that out of 500 participants, 19% had depression (Patient Health Questionnaire-9 [PHQ-9])) and 14% had anxiety (generalized anxiety disorder [GAD]) due to COVID-19 [20].

Moderate-or-severe anxiety was in higher proportion in females as compared to males, which is concurrent with the result of the study done by Devita M et al. in 2022, where being female and reporting the presence of subjective cognitive difficulties after COVID-19 were associated with higher levels of psychological distress [21].

According to the results of this study, it is clear that COVID-19 is a disorder associated with psychological distress, and PTSD is one of the common diagnoses, affecting nearly one-quarter of the population. To the best of our knowledge, none of the studies from India looked into the psychological status of admitted COVID-19 patients. However, few studies from other parts of the World reported 10% depression and PTSD one month after discharge, but they included only ten participants [22]. Another study reported that 16% and 13% of 294 patients reported probable PTSD, 29% and 20% probable anxiety, and 32% and 24% probable depression at one and three months after hospital discharge, respectively [14]. The current study is one of a kind, looking into the gray area of what happens psychologically to these patients when they are discharged from hospitals with a fair sample size.

A study by Putri DU et al. in 2021 reported that at least 16.5% of the sample had psychological distress upon hospital admission using a 5-item brief symptom rating scale, with distinct time-dependent decline [23]. This study also emphasizes the utmost need for proper and timely assessment and intervention. However, the impact of COVID-19 varies from individual to individual depending upon multiple factors ranging from individual personality type, co-morbidities, loss of family members, financial loss, or any other type of suffering. Thus, there should be a detailed assessment of the various factors that cause PTSD. Further, an in-depth analysis needs to be done to determine whether COVID-19 admission uniquely yields these PTSD symptoms or admission due to other illnesses also leads to PTSD.

The limitations of this study are that there was no sample size calculator used for the study; the duration of six months was taken into account. This was done as there was no inpatient study on COVID-19 then; moreover, any prediction could have hampered the data. It may be a possibility that this sample might not be representative. The study used only one scale, which assessed only one particular set of symptoms in detail. However, the subjects might be suffering from other psychiatric illnesses as well. The study evaluated only two factors, i.e., infection with COVID-19 and hospitalization, whereas this psychological impact could be multi-factorial.

## Conclusions

There is substantial evidence to suggest that COVID-19 has adversely affected the mental health of individuals. In this study, the majority of individuals infected with COVID-19 and admitted to the hospital suffered from significant psychiatric illness in the form of post-COVID-19 symptoms suggestive of PTSD, warranting the necessity of early requirement for pharmacological and non-pharmacological intervention by a mental health professional. Over half of the study subjects had severe PTSD symptoms, followed by those of a milder grade. In addition, as compared to males, females experienced more anxiety symptoms (p=0.002). Thus, it is recommended that timely non-pharmacological intervention be initiated, which might be significantly beneficial in reducing morbidity and improving the individual's quality of life.
